# VirTAXA: enhancing RNA virus taxonomic classification with remote homology search and tree-based validation

**DOI:** 10.1093/bioinformatics/btae575

**Published:** 2024-09-26

**Authors:** Yilin Zhu, Guowei Chen, Yanni Sun

**Affiliations:** Department of Electrical Engineering, City University of Hong Kong, Tat Chee Avenue, Kowloon, Hong Kong, 999077, China SAR; Department of Electrical Engineering, City University of Hong Kong, Tat Chee Avenue, Kowloon, Hong Kong, 999077, China SAR; Department of Electrical Engineering, City University of Hong Kong, Tat Chee Avenue, Kowloon, Hong Kong, 999077, China SAR

## Abstract

**Summary:**

RNA viruses are ubiquitous across a broad spectrum of ecosystems. Therefore, beyond their significant implications for public health, RNA viruses are also key players in ecological processes. High-through sequencing has accelerated the discovery of RNA viruses. Nevertheless, many of these viruses lack taxonomic annotation, posing a challenge to functional inference and evolutionary study. In particular, virus classification at the genus level remains difficult due to the limited reference data and ambiguous boundaries between some closely related genera. We introduce VirTAXA, a robust classification tool that combines remote homology search and tree-based validation to enhance the genus-level taxonomic classification of RNA viruses. VirTAXA is able to predict the genus label of an assembled viral contig and provide evidence type for each prediction. It achieves comparable accuracy to state-of-the-art methods while assigning genus labels to a greater number of sequences. Specifically, on the Global Ocean RNA metatranscriptomic data, VirTAXA can assign genus labels for 18% more contigs than the second-best classification tool. Furthermore, we demonstrated that VirTAXA can be conveniently extended to other types of viruses.

**Availability and implementation:**

The source code and data of VirTAXA are available via https://github.com/JudithEllyn/VirTAXA.

## 1 Introduction

RNA viruses, which contain ribonucleic acid (RNA) as their genetic material, include notable pathogens such as HIV, SARS-CoV-2, and influenza viruses. Research has consistently shown that many RNA viral pathogens possess zoonotic capabilities, initially residing in animal hosts before transitioning to infect human cells (Wolfe *et al.* 2007). Thus, obtaining a comprehensive RNA virus from various ecosystems is fundamental for emerging infectious disease surveillance and prevention.

High-throughput sequencing technologies have revolutionized the study of RNA viruses (Lipkin and Firth 2013). Several large-scale sequencing efforts covering the ocean, soil, humans, etc. (Xiong *et al.* 2020, Zayed *et al.* 2022) have significantly broadened our knowledge of the viral ecosystem and virus biology.

**Figure 1.**
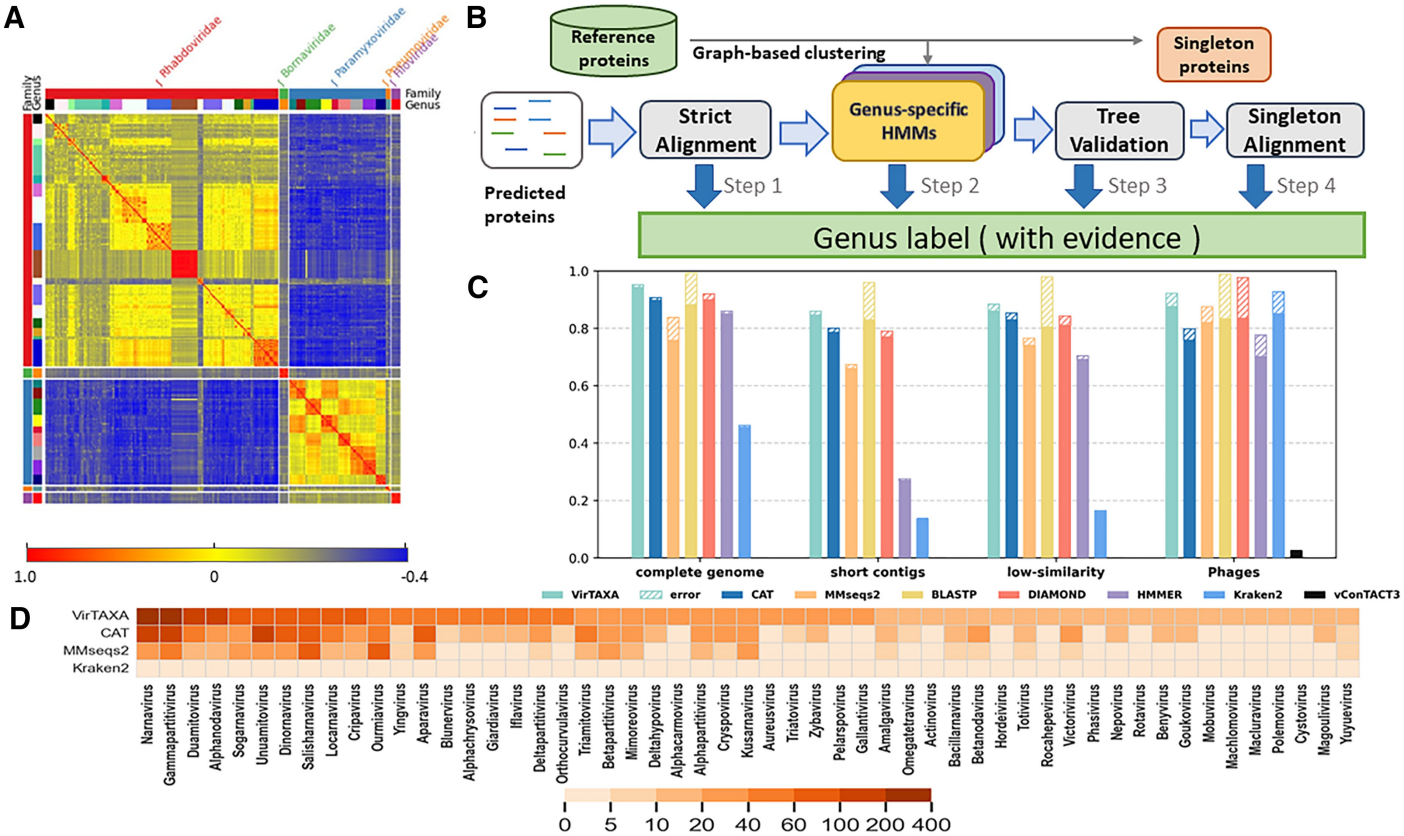
(A) Protein similarity heatmap for viral genomes in order *Mononegavirales*. Each cell in the heatmap represents the average protein sequence similarity [computed with BLASTP (Altschul *et al.* 1990)] between two virus genera (indicated by rows and columns). The color strips on the left and top depict the distinct separation of families and genera, respectively. (B) Sketch of VirTAXA. Cutoff of step 1: *E*-value < 1e−10, coverage*identity >50%. Step 2 and 3 are detailed in Section 2. Step 4: *E*-value < 1e−5, coverage >75%. (C) The experimental results of each tool on different scenarios, including the RefSeq complete genomes, simulated 500 bp contigs, low-similarity sequences and phage sequences. Each bar shows the percentage of classified sequences. In each bar, top shaded region shows the misclassification rate; bottom solid region represents the accuracy. (D) The heatmap visualization of the identified genera on GOV-RNA data. *X*-axis: genera containing >10 predicted contigs by at least one tool. *Y*-axis: four tools. Cell color intensity is proportional to the number of identified contigs in a genus by a tool.

Accurate taxonomic classification of viruses is of great significance because it facilitates the characterization of novel viruses. Based on the previous research (Yuan *et al.* 2022), the International Committee on Taxonomy of Viruses (ICTV) RNA virus taxonomy relies mainly on protein similarity comparisons and phylogenetic analyses at present. Despite promising results from generic and virus-specific taxonomic classification tools, the taxonomic classification of RNA viruses remains a nontrivial challenge. This is attributed to several factors. First, RNA viruses’ remarkable diversity necessitates employing highly sensitive homology search methods. Second, the representation of viruses within different genera is markedly skewed, reflecting a significant imbalance in the available viral sequences. Third, some genera share significant sequence similarity, leading to misclassifications when comparing viral sequences against the reference database. These properties can be partially visualized in Fig. 1A, which is a heatmap showing the protein-level inter-genera similarity.

As Fig. 1A shows, the families and the majority of genera exhibit substantial conservation at the protein level. However, some viruses, belonging to the same family but different genera lack distinct boundaries, such as those within the families *Rhabdoviridae* and *Paramyxoviridae*. Supplementary Fig. S1A presents the protein-sharing network between different genera. Some proteins are shared by viruses of different genera, requiring an optimized method for more accurate genus-level classification. More details about the challenges can be found in Supplementary Section S2.

## 2 Materials and methods

In this study, we developed a tool named VirTAXA for conducting genus-level classification of RNA viral contigs in metatranscriptomic data. It takes contigs as input, assigns the genus label, and provides the associated evidence. It can also recognize a new genus. Although genus label assignment can be conveniently formulated as a multi-classification problem in machine learning or deep learning, we choose to use the alignment-based method for two reasons. First, the long-tail distribution of the available genomes in all genera poses a significant challenge for learning-based models. Second, high-performance deep learning models have low interpretability, providing limited knowledge for biological discovery. In contrast, VirTAXA provides not only the classification but also the explainable evidence. We collected the complete RNA virus genomes released before 2023 from NCBI RefSeq as our reference sequences. In total, we have 5851 RNA virus genomes, including 106 families and 539 genera. VirTAXA contains four main steps by account of the ambiguous boundaries between some genera. Figure 1B sketches the main components of VirTAXA.

In the first step, VirTAXA classifies viruses with close relatives in the database. It assigns the genus label based on the best match from DIAMOND BLASTP (Buchfink *et al.* 2021). In the second step, VirTAXA assigns labels for remotely related viruses using profile Hidden Markov Models (pHMMs) search with customized cutoffs. For each genus, we conducted clustering for all the predicted proteins using MCL (Enright *et al.* 2002), resulting in 1658 clusters comprising at least 2 proteins (average cluster size of 6.3 members) total, and 3197 singletons. Then, we built pHMM for each cluster using HMMER3 (Eddy 1998). As these pHMMs are built using proteins in each genus, there is no guarantee that they will not incur statistically significant alignments against protein clusters in other genera. To screen genus-specific pHMMs and decide the best alignment score cutoff, we align the pHMMs of one genus against proteins of other genera. Here, we define two parameters, *pos* and *neg*, where *pos* denotes the lowest score generated by the protein members of the pHMM, and *neg* is the highest score generated by proteins from other genera. The adaptive cutoff was then computed as [neg+(pos−neg)*h], where *h* is a default value of 0.1. The difference distribution between *pos* and *neg* is illustrated in Supplementary Fig. S2. The majority of pHMMs exhibit a notable gap between their positive and negative scores, where a mere 22 out of 1658 pHMMs display higher negative scores compared to positive ones. In addition, 379 of these pHMMs lack any alignment with proteins from other genera, making them genus-specific pHMMs. Additionally, a high coverage threshold of 0.95 was used to ensure precise classification. Queries with pHMM alignments exceeding the adaptive cutoff or meeting the coverage cutoff were accepted as high-confidence predictions, while the ones falling below both thresholds (low confidence) will be further checked in the next step.

Then, we used phylogenetic trees to examine the predictions with low confidence from the previous step. Experimental analysis revealed that sequences from exogenous genera tend to be outliers in the phylogenetic tree, exhibiting significantly greater distances than the true members. To identify potential outliers, we employed TreeShrink (Mai and Mirarab 2018), a method that calculates a signature score for each sequence in the tree to detect an outlier. One example of tree-based validation is shown in Supplementary Fig. S3.

In the last step, we leverage the singleton references that cannot be clustered in the pHMM construction. The unclassified sequences from the previous steps will be aligned against the singletons by BLASTP with a relaxed cutoff. This step can classify viral sequences that are remotely related to those singleton proteins.

The prediction confidence decreases from the first to the last step. Thus, for each prediction, VirTAXA tells the user which step leads to this prediction, allowing users to decide whether to include all or part of the classification outputs. The detailed methodology and parameters are provided in Supplementary Section S3.1.

## 3 Results

We validated VirTAXA in various scenarios using both simulated and real sequencing data. In our comparisons, we benchmarked VirTAXA against six widely used tools: CAT (von Meijenfeldt *et al.* 2019), Kraken2 (Lu and Salzberg 2020), MMseqs2 (Mirdita *et al.* 2021), BLASTP, DIAMOND, and HMMER. They can perform genus-level classification with customized databases. All of the above tools were run with default settings and HMMER uses the optimized cutoffs designed in Section 2. It is also worth noting that these tools are not specifically designed for the classification of viruses. Although there exist several tools specifically designed for viruses, they cannot be utilized for benchmarking purposes due to either highly complex dependency errors or a lack of maintenance (see Supplementary Section S2.3). We report how many of the queries (test samples) can be assigned to a genus (i.e. prediction rate) and how many of these assignments are correct (i.e. accuracy). The detailed formula for the metrics can be found in Supplementary Section S3.2.

### 3.1 Performance on the complete genomes

To ensure an unbiased and thorough assessment of VirTAXA’s performance, all our experiments ensure that the test data has no overlap with the training sequences. In this experiment, we kept the genera containing >5 genomes in RefSeq, resulting in 5239 genomes. Then we used the first 60% of each genus as the reference data, containing 3218 sequences. Correspondingly, 1042 pHMMs were constructed from these reference sequences. The remaining 40% (2021) sequences constituted the test set.

The classification performance of the tools was visualized in Fig. 1C, where each bar represents the prediction rate and the solid region is the prediction accuracy. VirTAXA has a prediction rate of 95.1%, 4% higher than that of CAT. Meanwhile, our method achieves a high accuracy of 98.5%, slightly better than CAT (98.3%). On the other hand, Kraken2 demonstrates a lower prediction rate, which could be attributed to the high mutation rates observed in RNA virus sequences. Frequent mutations within RNA viral sequences make it difficult for Kraken2 to achieve exact matches, thereby hindering the establishment of specific taxonomic classifications. As VirTAXA contains four steps, the fractions and accuracy of the classification at each step of VirTAXA can be found in Supplementary Section S4.1.

### 3.2 Performance on the simulated short contigs

It is not rare that only part of the viral genome can be assembled by metatranscriptomic assembly programs. To evaluate the performance of the tools on short contigs, we created a hard case for classifying short contigs of 500 bp. We randomly cut 2021 500-bp contigs from the test set (same test set as Experiment 1). The results, presented in Fig. 1C, indicate that our method still achieved the best performance among these state-of-the-art tools from every metric. We have comparable accuracy to CAT and a 4.8% higher prediction rate. The contribution of each component of our tool is shown in Supplementary Section S4.1.

### 3.3 Performance on the low-similarity test set

In this experiment, we imitated the cases that assign taxonomic labels for diverged viruses. To achieve this objective, we created a complex case where the genomes in the test set exhibited low similarity with those in the training set following the approach as described in (Petti and Eddy 2022), which allows us to partition the viral genomes into two sets with specified inter-set similarity below 20%. Under this setting, the reference database comprised 1744 genomes, while the test set contained 1340 genomes. As shown in Fig. 1C, the low similarity test set impacted both the prediction rate and accuracy. VirTAXA achieved a prediction rate of 88.2%. While the accuracy of all the tools showed a decrease of 1%–2%, VirTAXA still achieved the best overall performance.

### 3.4 Handling open set problem

As new viruses can emerge with extensive sequencing efforts, a practically useful tool should be able to handle the “open set” problem, where viruses from new or unseen taxonomic groups are used as input. The classification tools should not classify these sequences into known taxonomic groups. To examine whether the tools can identify novel sequences, we applied VirTAXA on 636 sequences from families that were not part of the reference database as the novel query sequences. In this experiment, a lower prediction rate indicates a lower misclassification rate. The results show that BLASTP and DIAMOND misclassify a much higher proportion of these novel sequences (13.4% and 11.3%, respectively) compared to the other methods. VirTAXA and CAT both misclassified around 5% sequences in this experiment. This modest misclassification rate can be seen as the tradeoff for their high overall prediction rates.

### 3.5 Performance on the real sequencing dataset

We tested VirTAXA on the real dataset GOV-RNA (Zayed *et al.* 2022), which consists of 28 terabases (Tb) of Global Ocean RNA metatranscriptomic sequences. Specifically, we focused on 5504 virus operational taxonomic units (vOTUs) within the GOV-RNA dataset for our experiments. These vOTUs were defined based on criteria such as 90% average nucleotide identity over 80% coverage of the smaller contigs, with a minimum length of 1 kb. GOV-RNA is a real dataset without known genera labels. Using the total database (5851 genomes) as reference, the genus-level profiling results on this large dataset by different tools are shown in Fig. 1D. VirTAXA provides genus-level prediction for 2856 sequences, which achieved the highest prediction rate among the compared tools (CAT, MMseqs2, and Kraken2), consistent with the simulated data analysis findings.

Regarding sequences that cannot be classified into a genus, VirTAXA provides higher-level classification based on the alignment information (Supplementary Section S3.1.5). Among the unclassified sequences at genus level, there are 383 sequences predicted on family level, and 124, 236 sequences on order and class levels. Additionally, among the 861 RNA viruses classified at higher rank provided by the GOV-RNA authors (Zayed *et al.* 2022), VirTAXA can classify 85.3% of them with 92.8% consistent results, which is higher than CAT (predict 58.1% with 91.3% consistency), the second-best tool.

Furthermore, the sequences classified at the family level but not at the genus level may indicate new genera and thus exhibit as a branch in the phylogenetic tree. To introduce the emergent genus labels for the query sequences with unknown genera but assigned to the same family, we construct a phylogenetic tree for them with the reference sequences within that family. If a branch consists of >80% query sequences, we report this branch as a possible new genus. More details and examples about identifying possible new genera can be found in Supplementary Section S4.2. In 383 family-level predictions, 346 can form possibly new genera.

### 3.6 Adaptability of VirTAXA to other viruses

VirTAXA has exhibited its superior performance across multiple RNA virus datasets. Building upon these findings, we further investigate its applicability to other viruses. Thus, we assess VirTAXA’s extensibility on bacteriophages, mostly DNA viruses. As phage genomes are usually much longer than RNA viruses, we made corresponding changes in the genus assignment strategy, which can be found in Supplementary Section S4.3. Then we downloaded the complete bacteriophage genomes from the RefSeq database as a reference and utilized the testing datasets presented in the study by (Guan *et al.* 2023), which contains phage contigs cut randomly from the complete sequences. We added vConTACT3 (https://bitbucket.org/MAVERICLab/vcontact3/src/master/), one of the most popular DNA virus classification tools, for comparison. The results, shown in Fig. 1C, indicate that VirTAXA achieves a high prediction rate (92.2%) while maintaining a good accuracy (93.5%). Kraken2 performs better in classifying phage sequences due to the lower mutation rate in phages compared to RNA viruses. vConTACT3 did not perform well on these fragmented phage contigs.

## 4 Conclusion

In this study, we introduce VirTAXA, an RNA virus classification tool built on multiple methods, including pairwise sequence comparison, pHMM searching, and phylogenetic tree validation. We compare VirTAXA with several state-of-the-art tools using various RNA virus datasets with increasing complexity. The results demonstrate that VirTAXA achieves a higher prediction rate and comparable accuracy in classifying RNA viral sequences into genera than other tools. VirTAXA can accurately identify and classify recently found viruses (e.g. novel viruses). To assess the performance of VirTAXA in this regard, we conducted experiments using real metatranscriptomic data. The results show that VirTAXA can identify possibly new genera, highlighting its potential as a valuable resource for classifying new viruses and enhancing our knowledge of viral diversity. Furthermore, we explored the potential of extending VirTAXA to classify other virus sequences beyond RNA viruses.

However, we acknowledge that there is still room for further optimization and extension of VirTAXA in our future work. One area that demands attention is the enhancement of classification accuracy. Despite efforts, achieving 100% accuracy in genus-level classification remains a challenge. This is primarily due to the existence of certain sequences that bear close similarity to reference data from different genera, making them undetectable by the current validation method (Supplementary Section S4.4). Thus, there is a need to thoroughly evaluate these hard cases in our future work. Additionally, the determination of new genera in this study is considered preliminary and can be further improved. We aim to enhance the process of identifying new genera by incorporating more detailed features of the unclassified sequences into our classification framework. Finally, we plan to improve the efficiency of the pipeline. More analysis about the computational efficiency can be found in Supplementary Section S4.5. These efforts will contribute to the continuous development and enhancement of VirTAXA as a valuable tool for virus classification and analysis.

## Supplementary Material

btae575_Supplementary_Data
