# Supplementary material for: VirTAXA: enhancing RNA virus taxonomic classification with remote homology search and tree-based validation
Source: Bioinformatics. 2024 Sep 26;40(10):btae575. doi: 10.1093/bioinformatics/btae575 (PMC11464415; doi:10.1093/bioinformatics/btae575)
Supplement: btae575_Supplementary_Data [file btae575_supplementary_data.pdf]

VirTAXA: Enhancing RNA Virus Taxonomic Classification with remote homology search and tree-based validation

Yilin Zhu, Guowei Chen, and Yanni Sun

Supplementary materials

1 Summary

The supplementary file provides background information, detailed methods and result analysis of VirTAXA.

2 Challenges of Classifying RNA Viruses at the Genus Level

2.1 The characteristics of RNA viruses

RNA viruses face several key challenges mostly due to their distinct characteristics that set them apart from other types of viruses (Yuan et al., 2022). Firstly, many RNA viruses have relatively small genomes. This limited genomic information can make it harder to identify clear taxonomic markers that distinguish between viral taxa. Additionally, RNA viruses generally have higher mutation rates compared to DNA viruses, due to the lack of proofreading mechanisms in their RNA-dependent RNA polymerases. This high mutation rate leads to rapid genetic diversification even under the same genus. All of these factors pose challenges for RNA virus taxonomic classification, requiring more specific approaches that consider multiple genomic features and evolutionary relationships.

2.2 Inter-genus gene sharing is observed among multiple virus genera

We present a bipartite network to analyze the relationship between genera of RNA viruses, shown in Fig. S1 (A). The network encompasses two types of nodes: virus genera (colored nodes) and protein families (grey nodes). The protein families are constructed by clustering the protein sequences from all the underlying viral genomes in the RefSeq dataset using the Markov Chain

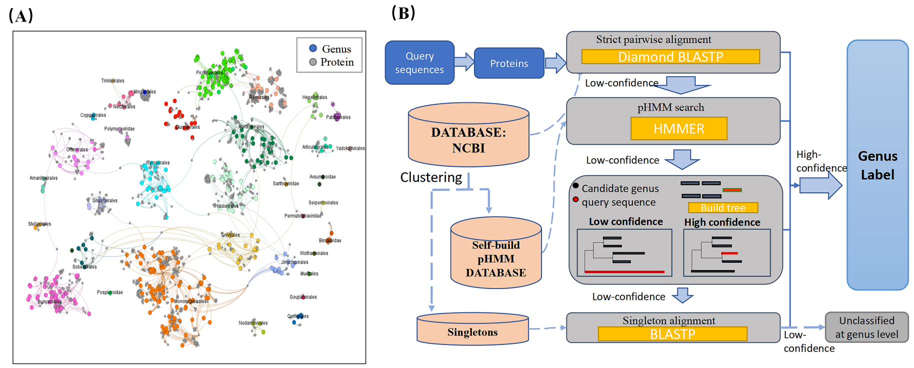

Fig. S1: (A): The network generated for derived protein families in viral genomes from the RefSeq database. The bipartite network is defined by protein families and genera. There are two types of nodes. Small gray dot: protein family. Large colored circle: genus. The color represents the order of the genus node. The protein family represents clustered protein sequences from all protein sequences derived from the viral genomes. An edge is drawn whenever a genus harbors a member of a protein family. (B): Detailed sketch of VirTAXA.

Clustering (MCL) algorithm (Enright et al., 2002). Due to the large number of genera, we use color to distinguish different virus orders. The figure reveals that viruses from distinct orders generally form subgraphs characterized by denser interconnections, and the inter-order connections are sparse. However, genera within the same order can still exhibit a higher frequency of connections to the same protein families, suggesting the presence of shared protein families among genera.

2.3 Limitations of commonly used taxonomic classification tools

Based on methodology, virus taxonomic classification tools can be roughly divided into three groups: alignment-based, short match-based, and learning-based.

Alignment-based methods encompass a range of tools including BLAST (Altschul et al., 1990), DIAMOND (Buchfink et al., 2021), VPF-Class (Pons et al., 2021), POGs (Pons et al., 2021), CAT (von Meijenfeldt et al., 2019), and MMseqs2 (Mirdita et al., 2021). BLAST does not scale well with fast accumulating metatranscriptomic sequencing data. DIAMOND has emerged to speed up the comparison and mitigate the computational burden by concurrently constructing indexes for both reference and query sequences. Utilizing sequence alignment programs, CAT and MMseqs2 can return the lowest common ancestor when there are ambiguous match results, which trade the resolution for accuracy. POGs provide a collection of orthologous gene clusters in

phages. Taxon-specific signatures within these clusters are used to align query sequences for the identification of virus taxa. VPF-Class provides taxonomic classification by comparing predicted proteins against the set of constructed Viral Protein Families (VPFs). Alignment-based tools generally achieve high precision (Zhu et al., 2022). However, relying solely on pairwise alignment can miss novel viruses that cannot be reliably aligned to reference sequences. Profile-based alignment methods can mitigate this issue to some extent. There are several tools that leverage protein profile HMMs as markers for different viral groups. For example, ClassiPhage (Chibani et al., 2019) employs a collection of profile HMMs that serve as taxonomic markers for the phage families *Myoviridae*, *Siphoviridae*, *Podoviridae* and *Inoviridae*. Similarly, the ViPhOG database (Moreno-Gallego and Reyes, 2021) provides a set of informative profile HMMs derived from viral proteins in the NCBI database, which can be used for viral classification. VIRIfy (Rangel-Pineros et al., 2023) is a recent pipeline allowing users to conduct prediction, functional, and taxonomic annotation of vial contigs using the informative pHMMs from the ViPhOG database. However, when using the profile-based methods, great care is needed in constructing protein families because they can be shared by different taxonomic groups and lead to ambiguous matches.

Short match-based tools include Kraken2 (Lu and Salzberg, 2020) and Kaiju (Menzel et al., 2016). Kraken2 utilizes k-mer matching, while Kaiju employs the minimum exact match to classify viral sequences. Short match-based methods tend to be highly efficient. However, they can incur false positive matches when k is too small or miss diverged viruses when k is too large.

There are also learning-enabled tools such as PhaGCN (Shang et al., 2021; Jiang et al., 2023), RdRpBin (Tang et al., 2022), CHEER (Shang and Sun, 2021), and vConTACTx (Bolduc et al., 2017; Zablocki et al., 2019) series. PhaGCN applies semi-supervised learning for bacteriophage classification, and PhaGCN2 extends this method to all viruses. RdRpBin utilizes both alignment-based and graph-learning methods to identify and classify reads. But both PhaGCN and RdRpBin can only achieve classification at the family level or above. CHEER conducts read-level taxonomic classification combining k-mer encoding and CNNs. vConTACTx employ a network-based approach and utilizes protein-sharing scores for virus classification. However, they have limited recall or classification rates for short contigs. And they suffer from highly imbalanced training samples from different taxonomic groups.

Totally, the above existing tools may find it challenging to classify RNA viruses. Alignment-based tools mostly rely on finding close homologs, often missing more distant evolutionary relationships, and leading to lower prediction rate. Similarly, short match-based tools perform poorly on RNA viruses, as their dependence on exact sequence matches is hindered by their high mutation rates, resulting in the inability to get hits. Even learning-based approaches struggle, as they typically require comprehensive reference databases and longer genomic sequences to extract stable taxonomic features - limitations posed by the small genome sizes and high mutation rate of RNA viruses. Therefore, there is a need to design an accurate and comprehensive tool specifically tailored for the

classification of RNA viruses at the genus level.

3 Material and Methods

While the paper sketches the main components in VirTAXA, a more detailed process can be found in Fig. S1 (B). In Step 1, the input queries are compared against the reference database using DIAMOMD within stringent criteria. Any viruses missed in this step are then aligned with pHMMs and assigned a "candidate genus" label in Step 2. For those with low confidence, a phylogenetic tree is constructed using reference sequences of the candidate genus for further examination. Finally, in the last step, the unclassified sequences from the previous steps are aligned against the singletons. Below we provide details about each step.

3.1 Technical Details of VirTAXA

3.1.1 Step 1: strict DIAMOND BLASTP

We collected the complete RNA virus genomes released before 2023 from NCBI RefSeq as our reference sequences. In total, we have 5,851 RNA virus genomes, including 106 families and 539 genera. To ensure high precision in the first step, we implemented a strict cutoff threshold (E-value<1e-10, query coverage*identity>50%), which is expected to achieve highly precise genus label predictions. In cases where a query sequence yielded multiple proteins surpassing the threshold, the alignment with the highest score will be selected. The imposition of a strict score cutoff ensured that only sequences exhibiting high similarity were considered for classification.

3.1.2 Step 2: pHMM search

In this step, we employed a systematic approach to build genus-specific pHMMs by utilizing RNA viral proteins from the reference database. To ensure optimal performance, we determined an adaptive cutoff value for each pHMM, which served as the threshold for the following sequence searches. After that, the constructed pHMMs were employed to accurately classify the query sequences, enabling robust and reliable analysis.

To build the pHMMs, we first predicted and translated genes from 5,851 sequences in the RefSeq database using Prodigal (Hyatt et al., 2010), resulting in 13,612 proteins. Then we performed pairwise alignments with DIAMOND under each genus, creating a graph where each node represents a protein sequence and the edge represents the alignment. Next, we conducted clustering using MCL, generating 1,658 clusters comprising more than one protein and 3,197 singletons. Each of the clusters comprises proteins from a single genus, and for each cluster, we conducted Multiple Sequence Alignment (MSA) using MAFFT (Katoh et al., 2002) and generated pHMMs by HMMER3 (Eddy, 1998).

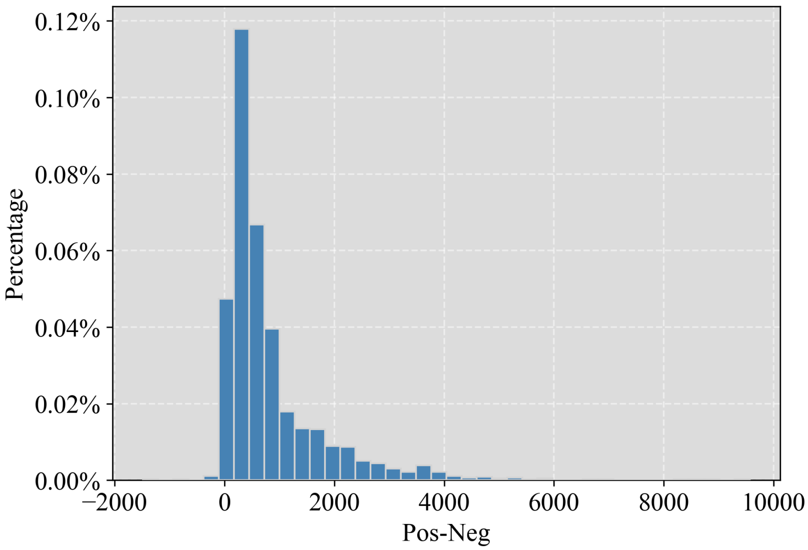

Fig. S2: The distribution of genus-distinguishing abilities of all pHMMs. We visualized the ability by calculating the difference in bit scores between member proteins (*pos*) and non-member proteins (*neg*) of the pHMMs. A small or even negative difference indicates that the pHMM may misclassify the genus of the query protein, suggesting the requirement of an adaptive cutoff.

Then we calculated the bit score cutoff for the pHMMs to facilitate the following classification matching process. Since each pHMM possesses a distinct conservation level, a fixed threshold is deemed inadequate. Thus, an adaptive cutoff was established for each pHMM by conducting hmmsearch against the reference sequences. Here, we define two parameters, *pos* and *neg*, where *pos* denotes the lowest bit score generated by the protein members of the pHMM, and *neg* is the highest bit score generated by proteins from other genera. The distribution of the difference between *pos* and *neg* for all 1,658 pHMMs is depicted in Fig. S2. The distribution reveals that the majority of pHMMs exhibit higher *pos* compared to *neg*, with a significant proportion falling within the range of 0 to 1000. This observation suggests that these RNA viral pHMMs can be considered genus-specific, making them suitable for conducting classification at the genus level. Furthermore, among the 1,658 pHMMs, 379 of them yield a *neg* of 0, which indicates that these particular pHMMs are more conserved as they fail to match with any proteins from other genera. Thus, the adaptive cutoff for these pHMMs is set at 0. In practical terms, once a query sequence aligns with any of these pHMMs, it will be assigned to a specific genus. For the remaining pHMMs, the adaptive cutoff values were computed as $[neg + (pos - neg) \times h]$, where h represents a default value of 0.1. This calculation ensures a balanced approach, taking into account both positive and negative scores to determine

the appropriate threshold for classification.

To classify virus genera, we aligned the protein of the sequences missed by step 1 against the pHMMs using hmmsearch, retaining the best-hit result. The associated genus label of the best-hit pHMM became the "candidate genus" for the query sequence. Classification accuracy was ensured by applying a stringent criterion using two metrics: HMM alignment bit score and coverage. Candidate labels exceeding the adaptive cutoff or meeting the coverage cutoff (95%) were accepted as high-confidence predictions, while the ones falling below both thresholds (low confidence) will be further checked in the next step.

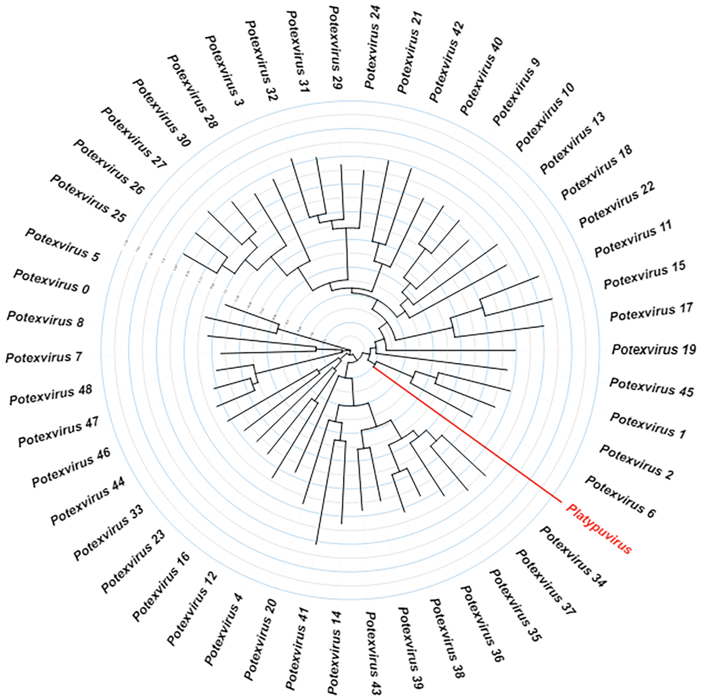

Fig. S3: A phylogenetic tree example. In this tree, the queried sequence, highlighted in red, originates from the genus *Platypuvirus*, which is distinct from its candidate genus represented in this tree, *Potexvirus*, while they are in the same family. It is evident that the query sequence exhibits a distinct outlier pattern, greater evolutionary distance, in comparison to the other sequences within the tree. All the phylogenetic tree figures presented were plotted using iTOL (Letunic and Bork, 2021).

3.1.3 Step 3: building phylogenetic trees

In this step, we used phylogenetic trees to examine the "candidate genus" with low confidence from the previous pHMMs. Experimental analysis revealed that

sequences from exogenous genera tend to be outliers in the phylogenetic tree, exhibiting significantly greater distances compared to sequences accurately assigned to their corresponding genus. An illustrative example is presented in Fig. S3, where a query sequence is placed into the phylogenetic tree of its candidate genus, while it originates from a different genus. The query exhibits the longest distance within the tree and thus is considered as an outlier within the context of this specific tree.

Specifically, for each input to this step, we built a phylogenetic tree using the query and the reference sequences in its candidate genus by FastTree (Price et al., 2010). Then we identify potential outliers with either TreeShrink (Mai and Mirarab, 2018) or the evolutionary distance of query (depending on whether the number of sequences meets the minimum requirement of TreeShrink). TreeShrink is a method that calculates a signature score for each sequence in the tree, which indicates the probability of being an outlier. Therefore, we reject the candidate genus of the query if it has the highest signature score or the longest distance from the root in the phylogenetic tree; otherwise, we recognize the candidate genus label. This process acts as a verification stage for the pHMMs' classification results and enhances the accuracy of the overall classification.

3.1.4 Step 4: singleton alignment

In this step, a relaxed cutoff is applied to the BLASTP alignment with the reference singleton sequences, with the e-value set to 1e-5 and query coverage set to greater than 75%. We only kept the best-aligned reference proteins over the threshold and assigned the genus label by the alignment. This step leverages the classification signal in the singleton proteins, thereby enhancing the comprehensiveness of the classification process.

3.1.5 Classification at higher levels

Although VirTAXA can predict more sequences at genus level than others, a substantial number of sequences still remain unclassified, indicating the significant divergence among the viruses. To provide more comprehensive taxonomic information, VirTAXA has included higher-level classifications for these unclassified sequences. The query sequences that could not be assigned at the genus level will be classified to higher taxonomic levels (Family, Order, Class, or Phylum) if they meet the cutoff criteria (E-value<1e-10 in DIAMOND or E-value<1e-10 and bit score>10% of adaptive cutoff in pHMM search). The higher-level classification will be based on the last common ancestor (LCA) of the top 'x' hits in both the DIAMOND and pHMM, where 'x' depends on the number of hits scoring higher than 50% of the best hit. With these improvements, VirTAXA can provide lineage information from genus to phylum levels. This extended classification capability allows VirTAXA to offer more comprehensive taxonomic information, particularly for sequences that cannot be confidently assigned at the lower taxonomic levels.

3.2 Performance metrics

To ensure a consistent comparison, we employed commonly used metrics to evaluate the classification performance of all the tools. The evaluation focuses on reporting how many of the inputs can be classified into a genus and how many of the assignments are correct. Correspondingly, these metrics included prediction rate $[N_{pred}/N_{all}]$ and accuracy $[N_{correct}/N_{pred}]$. The prediction rate was calculated by dividing the number of sequences with predictions (N_{pred}) by the total number of input sequences (N_{all}). The accuracy metric was determined by dividing the number of sequences with correct predictions $(N_{correct})$ by the number of sequences with predictions (N_{pred}). In the experiments, all the performances are evaluated at the genus rank.

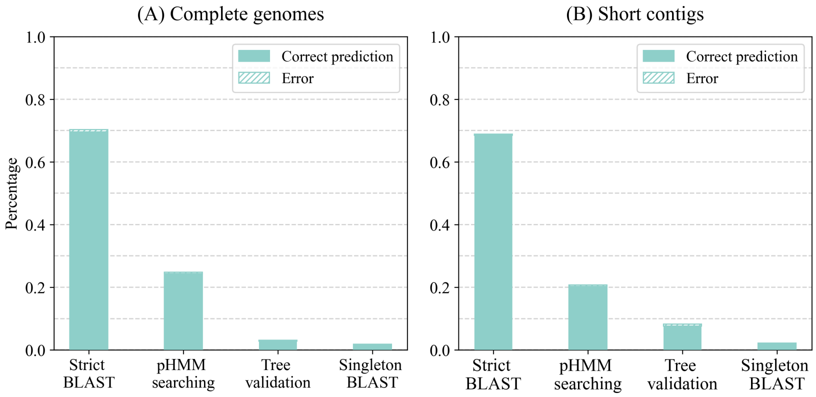

Fig. S4: The percentage of viruses that are classified at each step in VirTAXA. X-axis: the four steps. Y-axis: the percentage of input viruses classified at each step. (A): complete genomes; (B): short contigs.

4 Additional Results

4.1 Analysis of Each Step in VirTAXA

As VirTAXA involves 4 distinct steps, in our analysis, we also examined the classification result at each step of the process. Fig. S4 (A) illustrates the proportion of classified sequences in the complete genomes. Among the 1,921 sequences predicted by VirTAXA, 1,351 (70.3%) were identified in the first step using DIAMOND, and the accuracy in this step was 99%. The second step, employing pHMMs, directly classified 477 (24.8%) virus sequences with an accuracy of 98.1%. Additionally, 71 sequences underwent validation using phylogenetic trees due to lower bit score and query coverage, and 59 (3.1%) of them were kept, where the accuracy was 90%. Among the 12 sequences rejected by the phylogenetic trees, 7 of them were truly incorrect predictions. In the final

step, 34 (1.8%) sequences were classified by aligning against singleton sequences with an accuracy of 100%. The result reveals that the confidence of the pHMMs and the phylogenetic tree are lower than the strict DIAMOND BLASTP in the first step but can still achieve high accuracy.

Fig. S4 (B) presents the evidence distribution of each step on the simulated short contigs. Among the 1,725 contigs classified by VirTAXA, the distribution of sequences in each step is as follows: 1,189 (68.9%), 357 (20.7%), 141 (8.1%), and 38 (2.2%), respectively. In addition, the accuracy of each step is 99%, 98.3%, 92.2%, and 97.4%, respectively. Comparing these results to the previous experiment on complete genomes, the number of contigs predicted by strict DIAMOND BLASTP was barely affected. However, the prediction rate of pHMMs experienced a reduction, increasing contigs that needed validation through phylogenetic tree construction. The limited informative features on short contigs result in lower bit scores, which require tree-based validation when the scores fall below the threshold. These observations highlight the challenges posed by shorter sequences, as they require rigorous validation procedures to ensure an accurate result. Therefore, incorporating phylogenetic trees for the validation of low-confidence sequences, rather than outright rejection, proves to be quite beneficial.

4.2 The prediction of novel genera

VirTAXA provide a function to predict novel genera. In our methods, the sequences classified at the family level but not at the genus level may indicate new genera and thus exhibit as a branch in the phylogenetic tree. To introduce the emergent genus labels for the query sequences with unknown genera but assigned to the same family, we construct a phylogenetic tree for them with the reference sequences within that family. If a branch consists of more than 80% query sequences, we report this branch as a possible new genus. We designed this method based on the experimental analysis that sequences from the same genus tend to cluster together in distinct branches within the family tree (e.g., Family *Solemoviridae* in Fig. S5). When multiple sequences are grouped within the same branch, it indicates that these genomes share significant sequence similarity and are likely at a similar evolutionary stage. Thus, this can be interpreted as a potentially new genus-level cluster that is distinct from the references in the database.

To further validate the functionality, we conducted leave-one-genus-out experiments within the Family *Solemoviridae*. In each experiment, we left out sequences from one genus in the reference database (the same training set used in the complete genome experiment) and utilized the sequences from that genus as queries. We expected that VirTAXA would demonstrate that these queries belong to a potential new genus. Indeed, the output of VirTAXA indicated that all query sequences were unclassified at the genus level, receiving only a family-level label prediction (*Solemoviridae*). We then constructed a family-level phylogenetic tree using the query sequences and the sequences from the other genera. The three resulting trees shown in Fig. S6 revealed that the

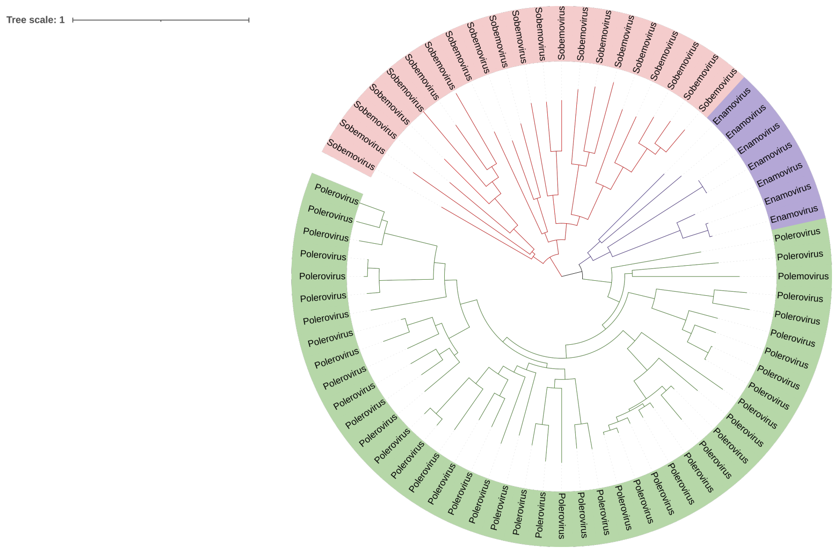

Fig. S5: The phylogenetice tree built by the sequences from Family *Solemoviridae* in the RefSeq database, where different colors represent different genera.

query sequences from the same "novel" genus tended to cluster together in a distinct branch. Therefore, it is reasonable to use this tree-based approach to effectively identify putative new genus-level clusters that are distinct from the existing reference database.

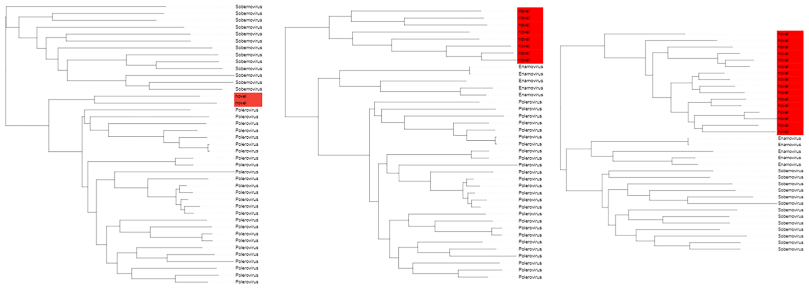

Fig. S6: The 3 phylogenetic trees constructed after the leave-one-genus-out experiments. The red part in each figure represents the genus that is left out.

However, it's important to note that this new branch cluster does not necessarily correspond to a strict standard genus unit. VirTAXA serves as a tool to identify potential novel viral lineages, but the biological significance and appropriate applications of these findings require further investigation and expert judgment. In future work, we aim to enhance the process of identifying new genera cluster by incorporating more detailed and robust features of the unclassified

sequences into our classification framework.

4.3 Adjustments for phage classification

Compared to RNA viruses, phages have different genome characteristics, including more genes in the genome, a lower mutation rate, and more homologous proteins shared by multiple taxonomic groups. Moreover, the taxonomic groups of phages are far from being complete because many new phages have not been sequenced or annotated yet. New genera can emerge with more extensive sequencing efforts. Drawing from these differences, we made some simple yet impactful adjustments to adapt VirTAXA to classify phage.

First, phage genomes are much larger than RNA viral genomes and contain more genes. It is more reasonable to consider all the proteins in the input sequence for classification. Thus, we employed the majority vote strategy based on the classification result of each protein. Second, we noticed more proteins aligned with multiple genera with high identities (e.g., 98%), which is rare in RNA viruses. These proteins are shared by multiple genera and can lead to ambiguous classification results. Thus, we collect all those proteins and exclude their results in the majority vote process. Finally, some of the pHMMs can be aligned with different genera with similar scores and E-values. Thus, we record those genera, and any alignment against these "ambiguous" pHMMs can lead to multiple labels, as it is hard to determine which genus is correct.

4.4 Analysis of the mis-classified prediction

To identify the potential improvement space, we analyzed the mis-classified cases in complete genomes and short contigs. Most of the misclassifications come across between closely related genera, such as between *Phlebovirus* and *Uukvirus* (under *Phenuiviridae*), and between *Mobavirus* and *Orthohantavirus* (under *Hantaviridae*). Then, we manually checked the misclassified sequences, and found that many of them have nearly identical gene fragments, which is the major reason for misclassification. For the *Mobavirus* sequences, the DIAMOND BLASTP alignment of the ORFs shows their coverage reaches nearly 100% to the sequences from the genus *Orthohantavirus*. This high similarity leads to misclassification between these two genera. To further improve the taxonomic classification, we will consider these special circumstances and try to merge these closely related genera as single labels for reference in future work. This approach is preferable to simply providing family-level predictions, as it can better account for the high similarity between specific genera that leads to misclassification.

4.5 Analysis on the runtime of VirTAXA

To evaluate the efficiency of VirTAXA, we recorded the total runtime of VirTAXA on classifying 2,000 complete genomes in the first experiment, which is 22min 23s, slower than that of CAT. The runtime for each of the 4 steps in the classification process are 2s, 2min 15s, 20min and 6s, respectively. The tree

validation step was found to consume the largest portion of the total runtime. The bottleneck is the construction of the multiple sequence alignment (MSA) before the tree building process. Although this phylogenetic validation step is computationally expensive, it helps improve the tradeoff between prediction rate and accuracy. VirTAXA can "save" some low-confidence query sequences that are rejected by other tools after building a phylogenetic tree for their candidate genus and found them not the outlier. From the performance, although this step takes extra time for VirTAXA, it can lead to a 4-5% higher prediction rate than that of CAT on the simulated sequences and more than 10% prediction rate on the real sequences. In order to improve the efficiency of VirTAXA, we provide a "faster" version that pre-builts some alignments of the genera in the database and added the query sequence to the existing alignments using the "add" method in MAFFT, which saves time and doesn't have a big impact on the results. This faster version only takes 4 minutes to run on the complete genomes above. In the future, we will continuously work on improving the running efficiency of VirTAXA.

References

Stephen F Altschul, Warren Gish, Webb Miller, Eugene W Myers, and David J Lipman. Basic local alignment search tool. *Journal of molecular biology*, 215 (3):403–410, 1990.

Benjamin Bolduc, Ho Bin Jang, Guilhem Doulcier, Zhi-Qiang You, Simon Roux, and Matthew B Sullivan. vcontact: an ivirus tool to classify double-stranded dna viruses that infect archaea and bacteria. *PeerJ*, 5:e3243, 2017.

Benjamin Buchfink, Klaus Reuter, and Hajk-Georg Drost. Sensitive protein alignments at tree-of-life scale using DIAMOND. *Nature methods*, 18(4):366–368, 2021.

Cynthia Maria Chibani, Anton Farr, Sandra Klama, Sascha Dietrich, and Heiko Liesegang. Classifying the unclassified: a phage classification method. *Viruses*, 11(2):195, 2019.

Sean R. Eddy. Profile hidden Markov models. *Bioinformatics (Oxford, England)*, 14(9):755–763, 1998.

Anton J Enright, Stijn Van Dongen, and Christos A Ouzounis. An efficient algorithm for large-scale detection of protein families. *Nucleic acids research*, 30(7):1575–1584, 2002.

Doug Hyatt, Gwo-Liang Chen, Philip F LoCascio, Miriam L Land, Frank W Larimer, and Loren J Hauser. Prodigal: prokaryotic gene recognition and translation initiation site identification. *BMC bioinformatics*, 11:1–11, 2010.

Jing-Zhe Jiang, Wen-Guang Yuan, Jiayu Shang, Ying-Hui Shi, Li-Ling Yang, Min Liu, Peng Zhu, Tao Jin, Yanni Sun, and Li-Hong Yuan. Virus classification for viral genomic fragments using PhaGCN2. *Briefings in Bioinformatics*, 24(1):bbac505, 2023.

Kazutaka Katoh, Kazuharu Misawa, Kei-ichi Kuma, and Takashi Miyata. MAFFT: a novel method for rapid multiple sequence alignment based on fast Fourier transform. *Nucleic acids research*, 30(14):3059–3066, 2002.

Ivica Letunic and Peer Bork. Interactive tree of life (itol) v5: an online tool for phylogenetic tree display and annotation. *Nucleic acids research*, 49(W1): W293–W296, 2021.

Jennifer Lu and Steven L Salzberg. Ultrafast and accurate 16s rRNA microbial community analysis using kraken 2. *Microbiome*, 8(1):124, 2020.

Uyen Mai and Siavash Mirarab. TreeShrink: fast and accurate detection of outlier long branches in collections of phylogenetic trees. *BMC genomics*, 19 (5):23–40, 2018.

Peter Menzel, Kim Lee Ng, and Anders Krogh. Fast and sensitive taxonomic classification for metagenomics with Kaiju. *Nature communications*, 7(1): 11257, 2016.

Milot Mirdita, Martin Steinegger, F Breitwieser, Johannes Söding, and E Levy Karin. Fast and sensitive taxonomic assignment to metagenomic contigs. *Bioinformatics*, 37(18):3029–3031, 2021.

Jaime Leonardo Moreno-Gallego and Alejandro Reyes. Informative regions in viral genomes. *Viruses*, 13(6):1164, 2021.

Joan Carles Pons, David Paez-Espino, Gabriel Riera, Natalia Ivanova, Nikos C Kyrpides, and Mercè Llabrés. VPF-Class: taxonomic assignment and host prediction of uncultivated viruses based on viral protein families. *Bioinformatics*, 2021.

Morgan N Price, Paramvir S Dehal, and Adam P Arkin. FastTree 2–approximately maximum-likelihood trees for large alignments. *PloS one*, 5 (3):e9490, 2010.

Guillermo Rangel-Pineros, Alexandre Almeida, Martin Beracochea, Ekaterina Sakharova, Manja Marz, Alejandro Reyes Muñoz, Martin Hölzer, and Robert D Finn. VIRify: An integrated detection, annotation and taxonomic classification pipeline using virus-specific protein profile hidden markov models. *PLOS Computational Biology*, 19(8):e1011422, 2023.

Jiayu Shang and Yanni Sun. CHEER: HierarCHical taxonomic classification for viral mEtagEnomic data via deep leaRning. *Methods*, 189:95–103, 2021.

Jiayu Shang, Jingzhe Jiang, and Yanni Sun. Bacteriophage classification for assembled contigs using graph convolutional network. *Bioinformatics*, 37 (Supplement_1):i25–i33, 2021.

Xubo Tang, Jiayu Shang, and Yanni Sun. Rdrp-based sensitive taxonomic classification of rna viruses for metagenomic data. *Briefings in Bioinformatics*, 23(2):bbac011, 2022.

FA von Meijenfeldt, Ksenia Arkhipova, Diego D Cambuy, Felipe H Coutinho, and Bas E Dutilh. Robust taxonomic classification of uncharted microbial sequences and bins with CAT and BAT. *Genome Biology*, 20(1):1–14, 2019.

Wen-Guang Yuan, Guang-Feng Liu, Ying-Hui Shi, Ke-Ming Xie, Jing-Zhe Jiang, and Li-Hong Yuan. A discussion of rna virus taxonomy based on the 2020 international committee on taxonomy of viruses report. *Frontiers in Microbiology*, 13:960465, 2022.

Olivier Zablocki, Ho Bin Jang, Benjamin Bolduc, and Matthew B Sullivan. vConTACT 2: A tool to automate genome-based prokaryotic viral taxonomy. In *Plant and Animal Genome XXVII Conference (January 12-16, 2019)*. PAG, 2019.

Yilin Zhu, Jiayu Shang, Cheng Peng, and Yanni Sun. Phage family classification under caudoviricetes: A review of current tools using the latest ICTV classification framework. *Frontiers in Microbiology*, 13:1032186, 2022.
